# Serotonin Coordinates Responses to Social Stress—What We Can Learn from Fish

**DOI:** 10.3389/fnins.2017.00595

**Published:** 2017-10-25

**Authors:** Tobias Backström, Svante Winberg

**Affiliations:** ^1^Institute of Integrated Natural Sciences, University Koblenz-Landau, Koblenz, Germany; ^2^Department of Neuroscience, Uppsala University, Uppsala, Sweden

**Keywords:** arginine vasotocin (AVT), corticotropin releasing factor (CRF), neurotransmitters, serotonin (5-HT), social behavior

## Abstract

Social interaction is stressful and subordinate individuals are often subjected to chronic stress, which greatly affects both their behavior and physiology. In teleost fish the social position of an individual may have long-term effects, such as effects on migration, age of sexual maturation or even sex. The brain serotonergic system plays a key role in coordinating autonomic, behavioral and neuroendocrine stress responses. Social subordination results in a chronic activation of the brain serotonergic system an effect, which seems to be central in the subordinate phenotype. However, behavioral effects of short-term acute activation of the serotonergic system are less obvious. As in other vertebrates, divergent stress coping styles, often referred to as proactive and reactive, has been described in teleosts. As demonstrated by selective breeding, stress coping styles appear to be partly heritable. However, teleost fish are characterized by plasticity, stress coping style being affected by social experience. Again, the brain serotonergic system appears to play an important role. Studies comparing brain gene expression of fish of different social rank and/or displaying divergent stress coping styles have identified several novel factors that seem important for controlling aggressive behavior and stress coping, e.g., histamine and hypocretin/orexin. These may also interact with brain monoaminergic systems, including serotonin.

## Introduction

The development of a dominance based social hierarchy is common among vertebrates, including teleost fish (Huntingford and Turner, 1987). The social rank of an animal has large effects on its behavior, physiology and life history trajectory (Fernald, 2003; Arnold and Taborsky, 2010; Ricci et al., 2013). In fact, this may be especially true for teleost fish with their complex life histories (e.g., Francis, 1992). Social subordination is an intense stressor and subordinate fish are subjected to chronic social stress and display elevated plasma cortisol, reduced growth, a general behavioral inhibition and other signs of chronic stress (Winberg et al., 2016). The effects of social stress has been mainly studied in the lab and the stress experienced by subordinate fish is obviously more intense in a confined environment, as in the lab or in an aquaculture setting, where they have limited possibilities to hide or escape (Sloman and Armstrong, 2002; Gilmour et al., 2005). Still, even in nature subordinate fish show signs of social stress (Sloman and Armstrong, 2002; Lema and Nevitt, 2004; Kline et al., 2011).

In general autonomic, endocrine and behavioral stress responses are well conserved and similarities between mammals and teleost fish are striking (Wendelaar Bonga, 1997; Gorissen and Flik, 2016). However, social stress sometimes has more pronounced effects in teleosts than in mammals, since in fishes reproductive functions and development is often controlled by social factors (Francis, 1992; Perry and Grober, 2003). For instance, in anadromous salmonid fish the age of smoltification varies within species, with faster growing fish smoltifying at an earlier age than those that are less competitive in growth (Klemetsen et al., 2003). In salmonid males, the ones with rapid initial growth may also become sexually mature at the parr stage, before leaving their natal river (Klemetsen et al., 2003). An even more dramatic effect of social interaction is sex reversal in teleosts that are sequential hermaphrodites (Francis, 1992; Godwin, 2010).

Both genetic and environmental factors play important roles in controlling life history traits, as well as intraspecific variance in behavioral and physiological traits, or what has been described as divergent stress coping styles (Koolhaas et al., 2007; Winberg et al., 2016). However, adjustments to the environment often require rapid, within generation, shifts in behavioral profiles. The winner/loser effect is a well-known example (Hsu et al., 2006; Oliveira et al., 2011). An animal that wins a fight for social dominance is more likely to win future fights even though the opponent may be larger and physically stronger. The loser on the other hand keeps on losing.

It is still far from clear how genes and environment interact in shaping behavioral phenotypes in teleost fish. However, the brain serotonergic system appears to be involved in these processes (Winberg and Thörnqvist, 2016). This review will focus on the role of serotonin in mediating different behavioral phenotypes in teleosts as well as the role of closely associated neuromodulators and neurotransmitters.

## The teleost serotonin system—similarities and differences compared to other vertebrates

**Figure 1:**
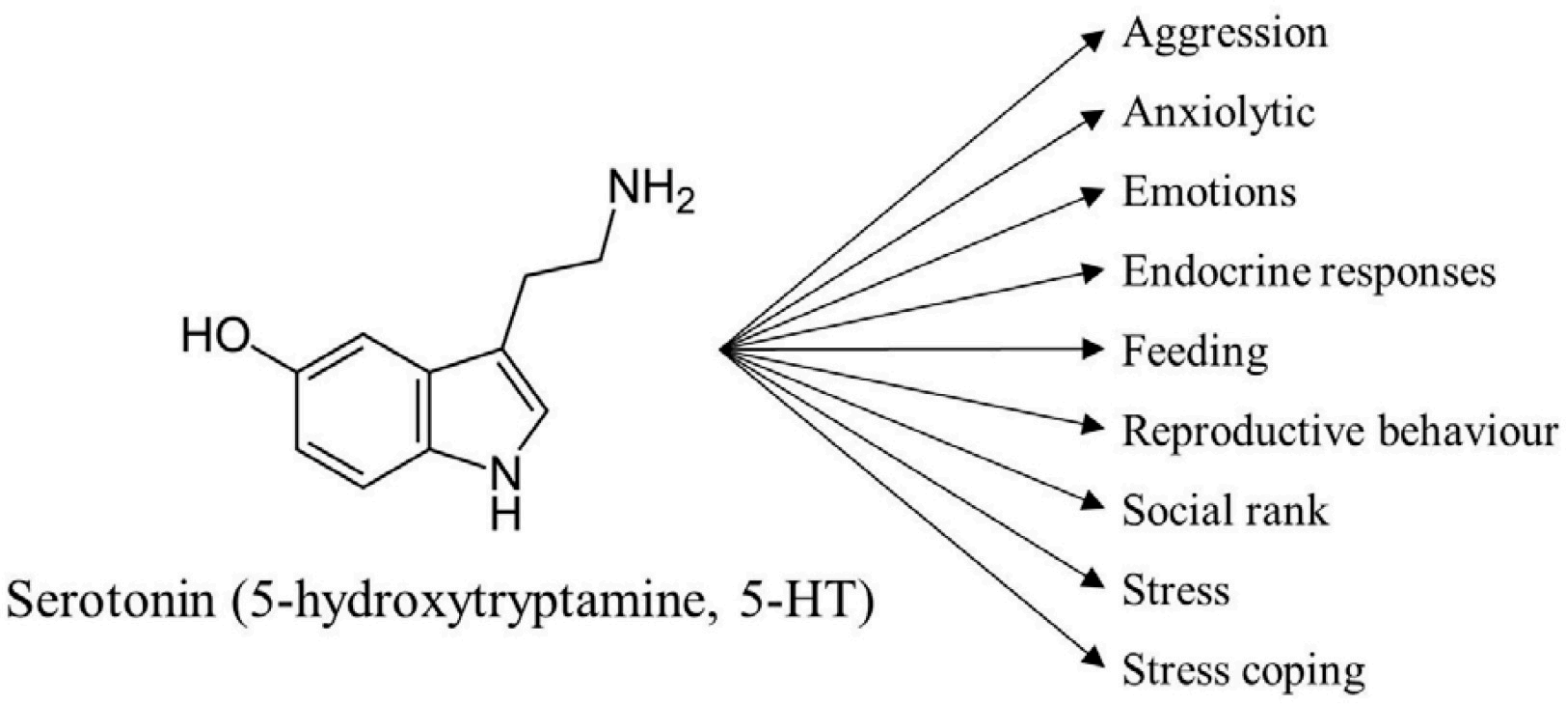
Serotonin has diverse and complex roles. Serotonin is involved in several different processes including, but not exclusively, these depicted here. Further information about the mentioned processes can be found in the article.

Serotonin (5-hydroxytryptamine, 5-HT) serves multiple functions in the vertebrate brain (see Figure 1), e.g., being involved in the control of emotions, endocrine responses, stress coping and aggression (Popova, 2006; Herculano and Maximino, 2014; Puglisi-Allegra and Andolina, 2015). Comparative studies have shown that 5-HT functions are highly conserved across vertebrates whereas 5-HT components and the general organization of the brain 5-HT system show some differences between teleost fish and amniote vertebrates (Lillesaar, 2011). In general the organization of the teleostean brain 5-HT system follows the general vertebrate pattern with 5-HTergic cell bodies mainly localized to the hindbrain, in the raphe nucleus. However, teleosts are characterized by having 5-HTergic cell bodies also in some other brain area, e.g., in the pretectum and basal forebrain (Lillesaar, 2011). The function of these 5-HT cell populations is still not known.

In zebrafish (*Danio rerio*), different paralogs of 5-HT specific genes are expressed in these different 5-HT populations. One example is the divergent expression of tryptophan hydroxylase (TPH) paralogs. This is the rate-limiting enzyme in 5-HT biosynthesis. The isoform TPH2, which has a Km for its substrate, L-tryptophan (Trp) that is close to the *in vivo* concentrations of Trp (McKinney et al., 2001, 2005), is expressed in the zebrafish raphe. Other TPH isoforms, TPH1a and TPH1b, are dominating in diencephalic and TPH3 in hypothalamic areas of the zebrafish brain (Ren et al., 2013). The kinetics of teleost TPH isoforms is not known but the zebrafish TPH3 appears to show kinetics comparable to those of mouse TPH1, which has a lower Km for its substrate Trp than TPH2 (Ren et al., 2013). Thus, in zebrafish as in mammals the rate of raphe 5-HT synthesis appears to be restricted by Trp availability. Consequently, the rate of raphe 5-HT synthesis will be affected by the amino acid composition of the diet and possibly also by sympathetic activation and immune responses (Russo et al., 2009). This opens the possibility that the amino acid Trp acts as a mechanism for the 5-HT system to monitor homeostatic challenges (Russo et al., 2009). By contrast, teleostean hypothalamic 5-HT neurons expressing TPH1 and TPH3 are probably less sensitive to changes in Trp availability. Still, Trp availability may affect hypothalamic 5-HT release through its effects on raphe 5-HT biosynthesis, even in teleosts (Lillesaar, 2011).

Serotonin acts on multiple 5-HT receptors and mammalian 5-HT receptors are divided into three families based on signal pathways. The 5-HT_3_ receptor is a cation specific ligand gated ion channel whereas 5-HT receptors belonging to the 5-HT_1_- and 5-HT_2_-receptor families are G-protein coupled, acting on G_i/o_ and G_q/11_, respectively. The 5-HT_2_-receptor family also includes the 5HT_4_, 5-HT_6_, and 5-HT_7_ receptors (Nichols and Nichols, 2008; Parsey, 2010). As a result of the teleost specific genome duplication (Le Comber and Smith, 2004) they show an even more striking 5-HT receptor divergence with multiple paralogous genes encoding specific 5-HT receptor subtypes. For instance, zebrafish expresses two 5-HT_1A_ receptor paralogs, the 5-HT_1Aα_ and 5-HT_1Aβ_ receptor, with divergent expression (Lillesaar, 2011; Horzmann and Freeman, 2016).

As for TPH, zebrafish express two paralogs of genes encoding serotonin re-uptake transporters (SERT, SLC6A4), slc6a4a (serta), and slc6a4b (sertb), again with divergent expression (Lillesaar, 2011; Horzmann and Freeman, 2016). The metabolizing enzyme monoamine oxidase (MAO), on the other hand, is only present as one form in zebrafish. In mammals, there are two forms, MAOA and MAOB, with different substrate specificity, MAOA being mainly responsible for 5-HT catabolism. Pharmacological experiments have shown that zebrafish MAO is more similar to the mammalian MAOA (Sallinen et al., 2009), even though it shares similar sequence homology with both mammalian MAOs (Anichtchik et al., 2006).

## Serotonin and stress—effects of social interaction

Losing fights for social dominance and being subordinate is highly stressful and has dramatic effects on the behavior and physiology of the individual. Typically, subordinate individuals show a behavioral inhibition, including appetite suppression, reduced aggression, and decreased reproductive behavior (Winberg and Nilsson, 1993; Wendelaar Bonga, 1997; Blanchard et al., 2001; Arregi et al., 2006; Bernier, 2006; Schreck, 2010). Subordinate fish in an established dominance hierarchy display a chronic activation of the brain 5-HT system (Winberg and Nilsson, 1993), a stress-induced effect that seems to be an important part of the mechanisms mediating behavioral inhibition (Winberg et al., 1993, 2001). However, the formation of a dominance hierarchy is stressful to both winners and losers and they both respond by a rapid activation of the brain 5-HT system but no sign of behavioral inhibition (Winberg et al., 1993; Øverli et al., 1999). This acute activation by the brain 5-HT system may in part be driven by an increase in brain levels of Trp (Winberg et al., 1993). As soon as the hierarchy is established brain concentrations of the 5–HT metabolite 5-hydroxyindoleacetic acid (5-HIAA) and 5-HIAA/5-HT ratios return to baseline levels in the dominant fish whereas these indexes of brain 5-HT activity remains elevated in subordinates even after long term social interaction (Winberg et al., 1993; Øverli et al., 1999). In fact, there is an inverse correlation between brain 5-HIAA/5-HT ratios and social rank of juvenile salmonids when kept in smaller groups with established hierarchies (Winberg et al., 1993). However, brain Trp levels tend to decrease, but still brain 5-HT concentrations of subordinates are usually retained at control levels. This suggests an up-regulation of brain 5-HT synthesis probably by increasing TPH activity, an effect that has been documented in mammals subjected to chronic stress (Chen and Miller, 2013). It is clear that long-term activation of the brain 5-HT system has an inhibitory effect on aggressive behavior (Winberg et al., 2001) but behavioral effects of an acute activation of the brain 5-HT system are less obvious.

Zebrafish (*Danio rerio*) is becoming an increasingly popular model also for studies on effects of agonistic interaction on behavior, stress responses, and brain monoamines (e.g., Dahlbom et al., 2012; Teles et al., 2013). Behavioral effects of social subordination are similar to those described in other teleosts, e.g., cichlids and juvenile salmonids (Oliveira et al., 2011; Dahlbom et al., 2012; Teles et al., 2013; Ivy et al., 2017). Behavioral effects of acute fluoxetine treatment has also been studied in zebrafish (Winberg and Thörnqvist, 2016). In this study, effects on 5-HT re-uptake were verified by analyses of brain 5-HIAA concentrations, and fluoxetine at both doses applied (immersing fish in 0.5 or 1.5 mg fluoxetine/l for 2 h) caused a significant decrease in 5-HIAA levels. However, the only behavioral effect observed was that fish treated with the lower dose spent more time in the center zone in an open field test, suggesting an anxiolytic effect of fluoxetine at this dose (Winberg and Thörnqvist, 2016). Acute fluoxetine at these doses had no effects on aggressive behavior. Thus, behavioral effects of chronic 5-HT activation are probably not directly mediated by elevated synaptic 5-HT concentrations but by secondary plastic changes in of the central nervous system.

## Serotonin interactions with the neuropeptides arginine vasotocin and corticotropin-releasing factor

Serotonin is an important modulator of social stress (as described above). However, some of these 5-HT related effects are mediated in concert with other neuromodulators. Especially, two neuropeptides are of interest based on their involvement in the stress axis, namely arginine vasotocin (AVT, the teleostean homolog of arginine vasopressin [AVP]) and corticotropin-releasing factor (CRF). Both are involved in the hypothalamic-pituitary-interrenal axis (HPI-axis, the teleostean homolog of the mammalian hypothalamic-pituitary-adrenocortical axis, where they stimulate pituitary release of adrenocorticotropin (ACTH) (Carrasco and Van de Kar, 2003). The role of AVT (Godwin and Thompson, 2012; Goodson, 2013) and CRF (Backström and Winberg, 2013; Hostetler and Ryabinin, 2013) have recently been reviewed concerning their involvement in social stress. Thus, this review will focus on the interactions between these neuropeptides and central 5-HT within teleost fish.

Interactions between AVT and 5-HT have not been thoroughly investigated in teleost fish. Still, there are some studies on effects of AVT on aggressive behavior and effects of 5-HT on AVT expression. For instance, Semsar et al. reported that treatment with fluoxetine reduces the expression of AVT mRNA in all cells of the preoptic area (POA) in male bluehead wrasse (*Thalassoma bifasciatum*) (Semsar et al., 2004). This was a follow-up to an earlier experiment were fluoxetine was shown to reduce aggression (Perreault et al., 2003), and thus indicating that aggression is affected by both AVT and 5-HT. Earlier reports in mammals have shown the same relationship between AVP and 5-HT (Delville et al., 1996). However, intracerobroventricular (ICV) injections of AVT in rainbow trout (*Oncorhynchus mykiss*) did not affect brainstem 5-HT neither in isolation nor under social interactions within 1 h (Backström and Winberg, 2009), but did increase serotonergic activity in the telencephalon and hypothalamus 8 h post-injection (Gesto et al., 2014). Thus, there are several indications that AVT and 5-HT are acting together modulating agonistic behavior in teleost fish.

Corticotropin-releasing factor and 5-HT interactions have been more thoroughly investigated, using different methods and concepts. The results show that CRF is involved in the control of several different behaviors in teleost fish. For instance, ICV injections of CRF increase locomotory activity in Chinook salmon (*Oncorhynchus tshawytscha*) (Clements et al., 2002). Similarly, downstream migration in Chinook salmon is increased after ICV injection of CRF (Clements and Schreck, 2004). Interestingly, this effect of CRF on locomotory activity is potentiated by a concurrent ICV injection of fluoxetine (Clements et al., 2003). This implies that CRF and 5-HT are interacting. In a later study, Carpenter et al. showed that ICV injection of CRF increased locomotion in rainbow trout (Carpenter et al., 2007). This CRF injection also increased 5-HT in the commissural nuclei of the ventral area of the telencephalon (subpallium) and raphe nuclei, and increased 5-HIAA in the hypothalamic preoptic area (POA) (Carpenter et al., 2007). Thus, in general the serotonergic activities in these areas were elevated and seemed to be associated with increased locomotory activity following CRF injections.

Further, CRF is involved in mediating behavioral effects of social interactions. For instance, it seems as if CRF can increase or decrease aggression depending on mammalian species (see reviews by Backström and Winberg, 2013; Hostetler and Ryabinin, 2013). Intererestingly, this has been seen in two studies of similar design in rainbow trout. Carpenter et al. reported that ICV injections of CRF suppressed attack behavior, and reduced latency to attack and retreat, but the ratio of attacks to retreats were increased and thus lead to dominance (Carpenter et al., 2009), whereas Backström et al. reported that CRF induced losing (Backström et al., 2011a). This difference could be attributed to the differing interaction times, 15 and 60 min respectively, as well as different methods for defining dominance. Interestingly, both of these studies investigated 5-HT as well. The study by Carpenter et al. in which 5-HT and 5-HIAA concentrations were assayed in specific brain regions, did not show any effects of CRF (Carpenter et al., 2009). However, in the study by Backström et al. ICV injections of the CRF antagonist α-helical-CRH_1−41_ decreased 5-HIAA and 5-HT in the brainstem (Backström et al., 2011a). This result suggests that CRF acts as a modulator of brainstem 5-HT activity. The interaction between CRF and 5-HT is also acting in the opposite direction since intravenous administration of the 5-HT_1A_ receptor agonist 8-OH-DPAT increased CRF mRNA in the hypothalamus of the Gulf toadfish (*Opsanus beta*) (Medeiros et al., 2014).

Another well-known effect of CRF is its appetite-suppressing effect in teleost fish (Bernier, 2006). Serotonin has also been reported to have appetite-suppressing effects, effects that may indicate an interaction between 5-HT and CRF. This suggestion is also supported by some studies in which fluoxetine treatment reduced food intake in goldfish (Mennigen et al., 2009, 2010). In these studies CRF seemed to increase in the anorexic effect in fluoxetine-treated fish (Mennigen et al., 2009, 2010). Further, Ortega et al. (2013) showed that the CRF antagonist α-helical-CRH_1−41_ if co-injected with 5-HT reversed the anorexic effect of 5-HT (Ortega et al., 2013).

## Serotonin and stress coping styles

During the last decades, inter-individual differences in behavior and/or physiology have been extensively studied. These differences have been termed differently depending upon discipline, e.g., behavioral syndromes (Sih et al., 2004), personality (Gosling, 2001) or temperament (Boissy, 1995). Stress coping style, which in addition to behavioral profile also includes physiological traits, is another frequently used term (Koolhaas et al., 2001, 2007). Two divergent stress coping styles referred to as proactive and reactive is usually distinguished (Koolhaas et al., 2001, 2007). Proactive individuals are typically bolder, more aggressive, and prone to form behavioral routines than reactive individuals. These are instead non-aggressive and shy and more plastic in their behavior. Further, in response to a stressor, proactive individuals display a lower HPI axis reactivity leading to a lower increase of plasma glucocorticoids than in reactive animals but a higher sympathetic reactivity than reactive animals. Stress coping strategies have been described in mammals (Koolhaas et al., 1999), birds (Cockrem, 2013), and teleost fish (Castanheira et al., 2015). In teleost fish, stress coping strategies have been reported in several species including halibut (*Hippoglossus hippoglossus*) (Kristiansen and Fernö, 2007), common carp (*Cyprinus carpio*) (Mackenzie et al., 2009), Senegalese sole (*Solea senegalensis*) (Silva et al., 2010), Nile tilapia (*Oreochromis niloticus*) (Barreto and Volpato, 2011), and zebrafish (Tudorache et al., 2015; Wong et al., 2015). Additionally, stress coping strategies have been found in several salmonid species including rainbow trout (Øverli et al., 2004), brown trout (*Salmo trutta*) (Brelin et al., 2005), Atlantic salmon (*Salmo salar*) (Kittilsen et al., 2009), and Arctic charr (*Salvelinus alpinus*) (Backström et al., 2014).

Serotonin is the neurotransmitter, which appears, most clearly involved in shaping the specific behavioral profiles of fish displaying divergent stress coping strategies, even though our knowledge on the exact role of 5-HT in mediating these effects are still limited. The importance of brain 5-HT in teleost coping strategies has been extensively studied in two rainbow trout strains selected for their stress responsiveness resulting in low (LR) and high (HR) responding strains (Pottinger and Carrick, 1999). For instance, serotonergic activity was higher in brainstem and telencephalon of LR compared to HR trouts (Øverli et al., 2001). This effect was evident both under non-stressed conditions (control) and after confinement stress in brainstem, but only under non-stressed conditions in telencephalon. Further, the serotonergic activity was higher in LR trout compared with HR trout after confinement stress for 1 and 3 h in both brainstem and telencephalon (Schjolden et al., 2006). However, in another study no difference was noted in the brainstem serotonergic activity following 30 and 180 min of confinement stress (Backström et al., 2011b).

Similar differences as the ones seen in the LR and HR trout have also been reported in other salmonid species. In Atlantic salmon sorted after emergence time from eggs (early and late being the first and last 25%), there was a difference in hindbrain serotonergic activity with late emerging salmons having a higher serotonergic activity (Thörnqvist et al., 2015). These early and late emerging salmon also differ in some behavioral characteristics (novel object and hypoxia) and are therefore proposed to be similar to stress coping strategies (Thörnqvist et al., 2015). Of further interest is that 5-HT1_Aα_ receptor mRNA levels are higher in early emerging salmon. Thus, there appears to be consistent differences between different stress coping strategies, but these differences are not apparent during all conditions.

## Social signals and stress—possible links with serotonin

Across vertebrates, melanin-based pigmentation has been associated with aggression, i.e., darker animals are more aggressive (Ducrest et al., 2008; Rushton and Templer, 2012). For instance, male Great Tits (*Parus major*) with a larger black (melanin-based) stripe area defended their territory more vigorously (Quesada and Senar, 2007) and male lions (*Panthera leo*) with darker manes are more aggressive (West and Packer, 2002). This pattern is also apparent in teleost fishes. For instance, an association between melanic coloration and aggression has been noted in *Gambusia* (Horth, 2003). The reason for this pattern is hypothesized to be based on the pleiotropic effects of melanocortin affecting both aggression and pigmentation (Ducrest et al., 2008). However, in this review we will focus on aggression and social stress and the possible association of 5-HT to changing signals and not these more or less permanent signals.

In an interesting contrast to the darker animals being more aggressive, several studies have shown that skin darkening signals subordination. For instance, in interacting dyads of Atlantic salmon the individual becoming subordinate also became darker in skin and eyes (O'connor et al., 1999). More aggression from the dominant fish leads to darker subordinate fish. This darkening appears to signal submission and aggression is withheld. Interestingly, this darkening happens faster between familiar salmons and thus acts to avoid an escalation of aggression (O'connor et al., 2000). Similarly, the salmonid Arctic charr also follow the general skin-darkening pattern for subordinates. After 5 days of interactions, the subordinate charr had darker skin (Höglund et al., 2000). Of special interest is that the subordinate fish also had higher serotonergic activity, as expected, and skin darkness was coupled with α-MSH (Höglund et al., 2000, 2002).

Pigmentation may also reflect individual stress coping styles and there are some indications that the melanin-based pigmentation can predict stress coping style. Having a high number of melanin-based spots indicates that the individual is proactive whereas having a low number of melanin-based spots indicates that the individual is reactive (Kittilsen et al., 2009). This relationship between skin pigmentation and stress coping has been observed in both Atlantic salmon and rainbow trout (Kittilsen et al., 2009). Similarly, proactive individuals of the convict cichlid (*Amatitlania siquia*) are darker although cortisol was not tested (Schweitzer et al., 2015).

**Figure 2:**
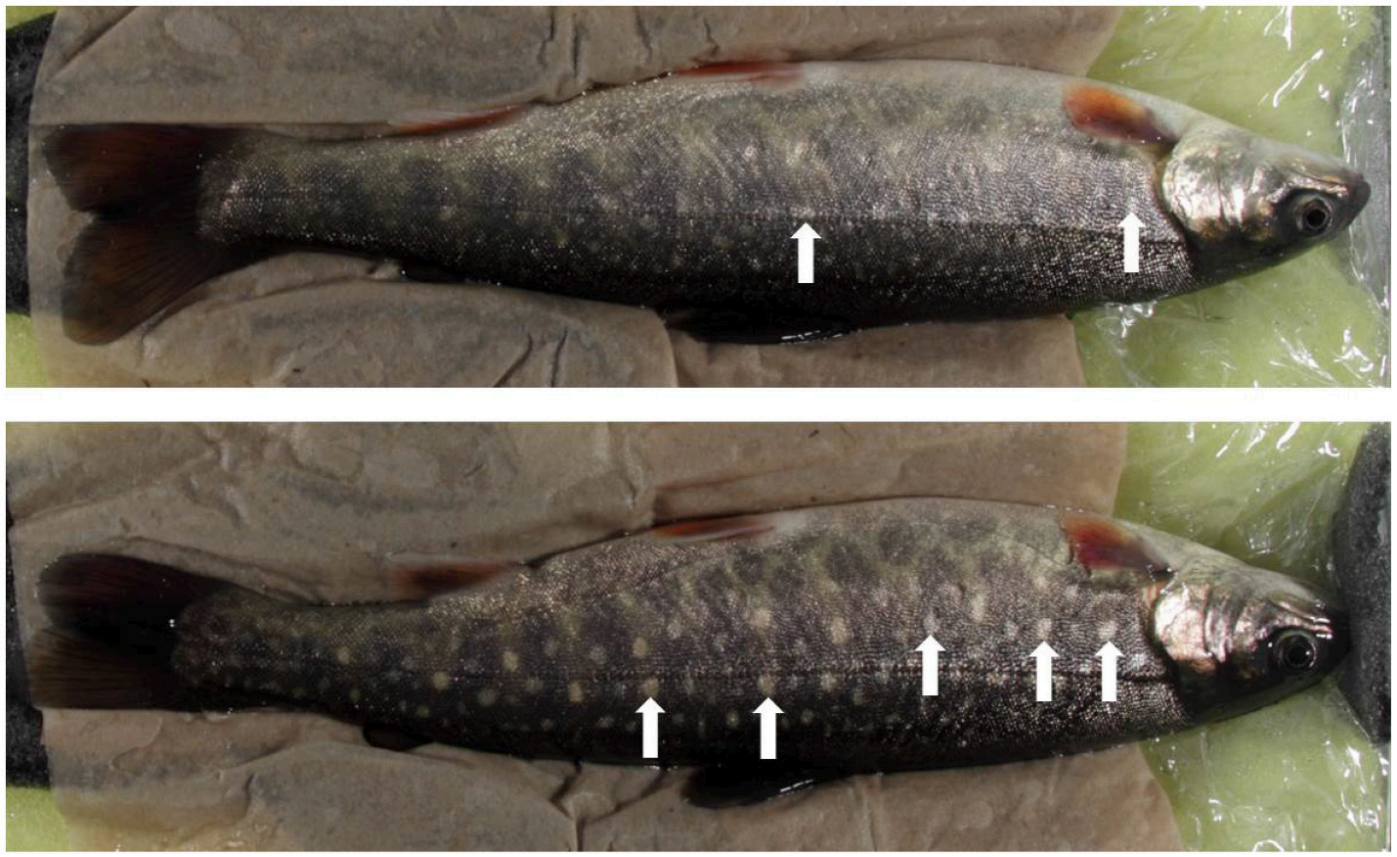
Carotenoid pigmentation in Arctic charr. Two photographs depicting the difference between an Arctic charr with few carotenoid-based spots (upper charr) and an Arctic charr with many carotenoid-based spots (lower charr). The white arrows indicate typical spots. In this example, the upper charr with fewer spots would represent a dominant and more stress resilient individual compared to the lower charr, which is subordinate and more stress sensitive. Further, there are also correlations between spots and serotonin (see text for further details).

Further, the associations between carotenoid-based pigmentation (instead of melanin-based), social signaling and stress have recently been investigated in Arctic charr (see Figure 2). Skin pigmentation of Arctic charr, and other species of the genus *Salvelinus*, differs from other salmonids (Shahidi et al., 1994; Klemetsen et al., 2003). In Arctic charr, it was shown that carotenoid pigmentation could predict stress responsiveness with less spots indicating a more stress resilient individual (Backström et al., 2014). Secondly, after dyadic encounters dominant individuals had fewer spots than subordinate individuals (Backström et al., 2015a). Thirdly, in a study covering over a hundred families of Arctic charr it was shown that carotenoid pigmentation was a heritable trait (Nilsson et al., 2016). Thus, it seems likely that in Arctic charr carotenoid-based pigmentation is important for social communication, and it seems as if the changes are induced quickly (within 2 min) (Backström et al., 2015b). Further, serotonergic activity differed between dominant and subordinate charr (Backström et al., 2015c) as expected and as earlier reported. In dominant individuals, telencephalic 5-HIAA/5-HT was positively correlated to spots, and in subordinate individuals, optic tectum 5-HIAA was negatively correlated to spots (Backström et al., 2015c). Similarly, in another experiment on the effects of anesthetics both optic tectum 5-HIAA and telencephalic 5-HT were correlated to spots, positive and negative respectively, in two different treatment groups (Backström et al., 2017). Thus, carotenoid pigmentation in Arctic charr is associated with stress responsiveness and stress coping styles, similar to the case of melanin pigmentation in Atlantic salmon and rainbow trout. This connection between social signals, stress and 5-HT need further investigations within other species, especially outside the salmonids.

## Studies on brain gene expression

As discussed above, the brain monoaminergic systems are together with cortisol and the neuropeptides of the HPI axis (e.g., AVT and CRF) major players responsible for shaping the behavioral profile of individual fish according to its social experience. However, behavioral effects of social interaction are complex, including effects on aggression, boldness, arousal etc. Thus, multifactorial control mechanisms are to be expected. Moreover, effects of social interaction may vary between individuals depending on genotype, context and previous experience (Koolhaas et al., 2007). Thus, not surprisingly studies on brain gene expression have identified numerous genes that are differentially expressed when comparing fish of different social status, or fish displaying divergent stress coping styles. Differentially expressed genes include genes encoding various components of the brain monoaminergic systems, e.g., enzymes, receptors and re-uptake pumps (Mackenzie et al., 2009; Aubin-Horth et al., 2012; Thörnqvist et al., 2015; Rey et al., 2016). In addition, the expression of numerous genes related to neuronal plasticity has been reported to differ depending on social status and/or coping style (Teles et al., 2016). Several novel mechanisms mediating effects of social interaction have been identified in gene expression studies. For instance, recently RNA-sequencing was used to identify transcriptome differences between a proactive and reactive strain of zebrafish (Wong et al., 2015). Over 1900 genes differed in expression between the two strains, and a more specialized and conservative analysis showed that 62 genes differed in expression between the two strains and these included GABAergic, non-apeptidergic, and glucocorticoid pathways as have previously been described. However, this study provides evidence for the existence of several other pathways involved in or affected by stress coping strategies. Thus, this is a rapidly advancing field of research and it is not our aim to provide a comprehensive review. Instead we will focus our discussion on just a few studies where effects on brain gene expression could be related to the 5-HT system and the mechanisms discussed in the present review.

One such study is the one by Filby et al. (2010). In that study the expression of 40 genes, representing key components of major transmitter systems and neuroendocrine mechanisms, were quantified in four different brain regions of dominant and subordinate male and female zebrafish. The results show that no less than 34 of these genes are differentially expressed and that telencephalon and hypothalamus are the brain regions showing highest number of differentially expressed genes. Differentially expressed genes include genes of the 5-HT, dopamine (DA), AVT, isotocin and somatostatin pathways as well as genes coding for key components of the HPI axis and the hypothalamic-pituitary-gonadal axis. In most cases these pathways have previously been implicated in the control of aggressive behavior in fish (Filby et al., 2010).

However, in addition to these genes Filby et al. (2010) also reported that genes of the histamine system, i.e., histidine decarboxylase (HDC) and histamine receptor 2, were differentially expressed in dominant and subordinate zebrafish. The study by Pavlidis et al. (2011) reported a similar elevation of HDC mRNA expression in dominant male zebrafish, confirming the results by Filby et al. (2010). In addition, Pavlidis et al. (2011) reported a similar up-regulation of hypocretin/orexin (Hcrt) expression in the brain of dominant male zebrafish.

Arousal and wakefulness are controlled by Hcrt through its stimulatory action on the aminergic systems, i.e., 5-HT, DA, norepinephrine, acetylcholine and histamine (Chieffi et al., 2017). Thus, together these systems form a complex neuronal network promoting wakefulness and arousal. However, in addition to controlling circadian sleep wake cycles Hcrt plays an important role in the control of feeding behavior, energy homeostasis, reward systems, cognition and mood (Chieffi et al., 2017). Similarly, the brain histaminergic system clearly has functions beyond sleep control. In mammals, brain histamine appears to be involved in learning, memory consolidation, anxiety, locomotion, feeding and neuroendocrine regulation (Panula and Nuutinen, 2013). Histamine mediates these effects through interaction with other neurotransmitters, including the 5-HT system. These widespread neuromodulatory actions of the orexigenic and histaminergic systems makes them very interesting candidates for mediating effects of social experience on behavioral responsiveness.

Interestingly, zebrafish with a mutation in the gene encoding fibroblast growth factor receptor 1 a (fgfr 1a), also known as spiegel danio, is bolder and show elevated aggressive behavior as compared to wild type fish (Norton and Bally-Cuif, 2010). The fgfr1 a mutation affects brain development, one major effect being a reduction in brain histamine. The fact that pharmacological elevation of histamine signaling restores normal behavior in the mutant fish made the authors suggests a role of brain histamine in the control of aggressive behavior and boldness in zebrafish (Norton and Bally-Cuif, 2010). However, the fgfr 1a mutation also had effects on the brain 5-HT system. The mutants showed higher numbers of neurons expressing the 5-HT transporter gene slc6a4a (serta) in the raphe. Thus, the fgfr1a mutation seems to affect the development of the histaminergic as well as the 5-HTergic system. Even though, acute fluoxetine did not restore normal behavior in mutant fish an involvement of the 5-HT system cannot be excluded.

## Conclusions and future perspectives

This review has focused on the role of brain 5-HT in coordinating behavioral and neuroendocrine effects of social stress in teleost fish. The brain 5-HT system is clearly central in mediating effects of social stress. These effects of elevated brain 5-HTergic activity appear to be mediated mainly through neuroplastic responses, in some cases including interactions with other neuromodulators as for example the peptides AVT and CRF. Immediate effects of an acute activation of the brain 5-HT system are less obvious. Serotonergic functions seem to have been highly conserved throughout the vertebrate subphylum. Still, the anatomical organization of the teleost 5-HT system differs from that of mammals and functional correlates of these differences have not been addressed in any detail.

Divergent stress coping styles are at least in part controlled by heritable factors. Clearly, genes and environmental factors interact together controlling behavior and neuroendocrine stress responses. Moreover, and perhaps most interesting but less studied, is if the effects of social environment will differ depending on heritable factors. These are questions that could be addressed by selection experiments, generating fish strains displaying divergent coping styles. Fish from these strains could be subjected to different environmental challenges and the response compared. Zebrafish, having short generation time and well-developed molecular tools, holds a great potential as a model species in such studies. A better understanding on these classical questions (i.e., phenotypic plasticity and the gene environment interaction) is important for our understanding of how divergent life history trajectories are controlled. It is also highly relevant for welfare and applied research on aquaculture. Effects of social interaction in zebrafish also provide a useful and relevant model for biomedical studies on drug addiction and affective disorders in humans.

## Author contributions

All authors listed have made a substantial, direct and intellectual contribution to the work, and approved it for publication.

### Conflict of interest statement

The authors declare that the research was conducted in the absence of any commercial or financial relationships that could be construed as a potential conflict of interest.
